# Quantum Molecular Dynamics Approach to Understanding Interactions in Betaine Chloride and Amino Acid Natural Deep Eutectic Solvents

**DOI:** 10.1021/acsphyschemau.4c00072

**Published:** 2024-11-09

**Authors:** Eudes Eterno Fileti, Henrique de Araujo Chagas, Guilherme Colherinhas, Thaciana Malaspina

**Affiliations:** † Instituto de Ciência e Tecnologia, Universidade Federal de São Paulo, São José dos Campos, 12247-014 São Paulo, Brazil; ‡ Instituto de Física, Universidade Federal de Goiás, 74690-900 Goiânia, GO, Brazil

**Keywords:** AIMD, natural deep eutectic solvents, amino acids, hydrogen bonds, proton transference

## Abstract

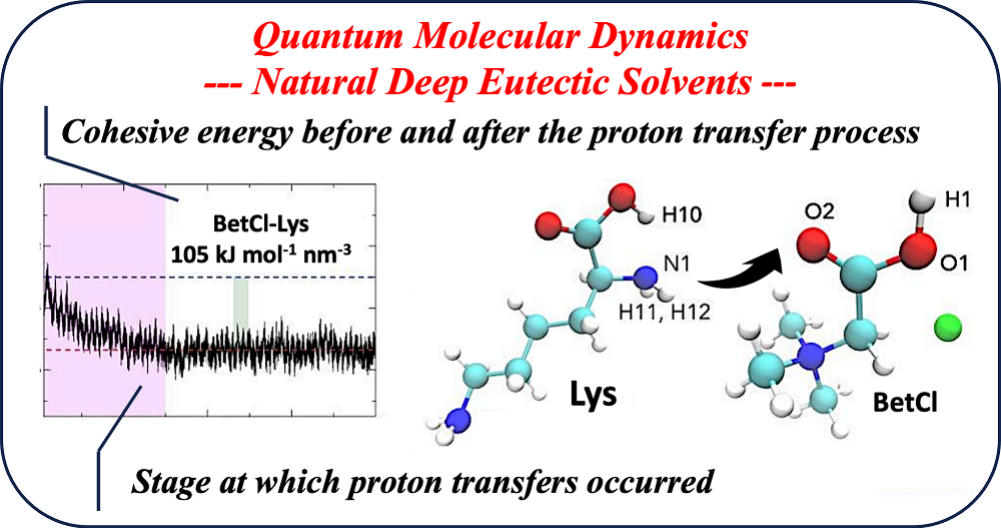

The unique properties and versatile applications of natural deep eutectic solvents (NaDES) have sparked significant interest in the field of green chemistry. Comprised of natural components that form liquids at room temperature through strong noncovalent electrostatic interaction, these solvents are cost-effective, nontoxic, and versatile. Betaine chloride-based NaDES, in particular, have shown promise in biocatalysis and sugar extraction due to their excellent properties. Despite their potential, the complex nature of these solvents, characterized by intense hydrogen bonding and proton transfer processes, poses significant challenges. This study employs quantum molecular dynamics (ab initio MD-AIMD) to explore the intricate NaDES-microstructure formed from betaine chloride and amino acids (arginine, histidine, lysine). Our findings highlight the dynamic nature of proton transfers within these solvents, demonstrating rapid and extensive hydrogen bonding interactions. The Van Hove correlation functions reveal that proton transfers are highly mobile, facilitating the formation and breaking of covalent hydrogen bonds. This dynamic behavior is further corroborated by the radial distribution functions, which indicate significant proton exchange between amino acids and betaine cations. Chloride anions play a crucial role in maintaining the structural integrity of NaDES through strong interactions with proton donors. These findings advance our understanding of these eutectic solvents and their potential applications in sustainable chemical processes.

## 1 Introduction

Natural Deep Eutectic Solvents (NaDES) have become a groundbreaking class of green solvents, recognized for their unique physical properties and extensive technological applications.^1−4^ Due to their biocompatibility, stability, and dense hydrogen bond networks, NaDES are well-suited for biocatalysis, enhancing enzyme stability and reaction efficiency. In pharmaceuticals, they offer improved solubility for hydrophobic drugs and better biodistribution, acting as green solvents with minimal toxicity. Moreover, NaDES provide sustainable alternatives in green chemistry, reducing waste and eliminating harmful solvents while supporting efficient, environmentally friendly reactions. These unique properties underscore the broad applicability of NaDES in biocatalysis, pharmaceuticals, and sustainable chemical processes.^1−4^

These solvents, composed of natural origin components, are mixtures of two or more compounds that, when combined in specific molar ratios, exhibit a substantial depression in their melting points, often becoming liquid at room temperature.^3−5^ This phenomenon results from the formation of strong intermolecular hydrogen bonds (HB) between the components, one component functions as a hydrogen bond donor (HBD) while the other serves as a hydrogen bond acceptor (HBA).^5,6^ The preparation of NaDES is straightforward, typically involving physical mixing, which can be expedited through heating and vigorous agitation. The use of readily available natural materials, such as sugars, alcohols, and organic acids, makes NaDES cost-effective and safer, minimizing toxicity risks for workers in industrial and end-users.^1−4^

Recently, NaDES based on betaine chloride have garnered significant attention.^7−12^ Betaine (Bet) is an affordable, naturally derived compound featuring a quaternary trimethylalkylammonium group and a carboxylate group, notable for its biodegradable and nontoxic properties.^9^ Betaine-based NaDES have proven suitable as highly efficient cosolvents in biocatalytic reactions, functioning as protective agents against denaturation and substantially increasing activity.^10^ The potential of betaine-based NaDES for the extraction of reducing sugars was explored by Banat and colleagues,^11^ based on criteria that consider their excellent balance of properties of interest, such as toxicity, viscosity, density, and solubility.

Investigating NaDES presents significant challenges due to their complex nature, characterized by long relaxation times resulting from strong hydrogen bonds, intense polarization effects, and proton transfer processes.^1−3,8^ Classical Molecular Dynamics (CMD) simulations have been effective in studying the thermophysical properties of Deep Eutectic Solvents (DES), such as diffusivity, viscosity, and ionic conductivity.^13^ However, this classical method is inadequate for accurately describing certain solvent properties, especially those involving polarization and chemical reactivity. In such cases, we use AIMD, which allows for the study of specific interactions, such as hydrogen bond formation and the prediction of charge/proton transfer, as well as accurately predicting polarization effects and dynamic charge distributions. AIMD provides a detailed electronic structure that accurately describes polarization and reactivity.^14^

Despite the increasing number of publications on betaine-based NaDES, information on the dynamics of its complex protic network of noncovalent electrostatic interactions is limited. This study investigates three natural deep eutectic solvents based on betaine chloride and three amino acids (AA): arginine, histidine, and lysine. AAs, being abundant in nature, are excellent feedstocks for NaDES synthesis.^1−4^ To address the challenges posed by the nature of these solvents, we employ AIMD simulations, a technique that describes the dynamic evolution of electron density in a way that allows us to accurately characterize the solvation environment at the atomic and electronic levels and capture both polarization effects and possible proton transfers from the esthetic solutions. We aim to delve into the microstructure of these DESs, with a particular focus on the intricate noncovalent electrostatic interactions among the various species involved.

## 2 Methods

Before discussing the protocol, it is important to distinguish between betaine and betaine chloride, which are related but have significant structural and functional differences. Betaine, also known as trimethylglycine, is a *zwitterionic* compound with both positive and negative charges on the same molecule, this makes it an ideal HBA, as it does not engage in strong ion–ion interactions with itself.^9^ Betaine can form DES with organic substances and it can donate HB and is considered a nonselective and universal HBA for DES formation.^10−12^ In contrast, betaine chloride is a protonated form of betaine associated with a chloride anion, which can act as HBD and HBA. Like betaine, it is water-soluble and has been used to produce DES, although it creates a more acidic chemical environment. To make it clear, this study will investigate choline chloride-based liquids, bearing in mind the possibility of choline chloride deprotonation and partial conversion to betaine.

**Figure 1:**
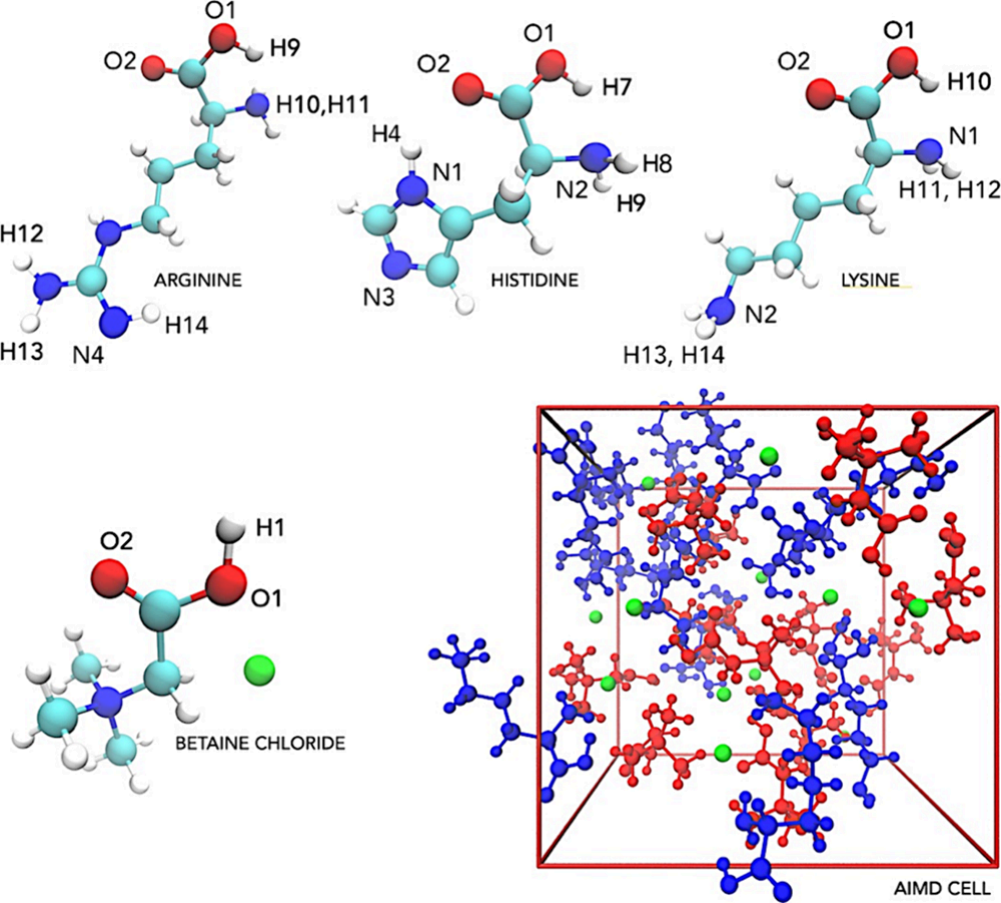
Molecular configurations for the four solvent components: arginine, histidine, lysine, and the ion pair betaine chloride. A representative equilibrated computational ab initio molecular dynamic simulation cell (AIMD, with 517 atoms) for the BetCl-Arg eutectic solution is shown, where chloride, betaine, and arginine are denoted in green, red, and blue, respectively. For clarity, hydrogen atoms are omitted in simulation cells.

In this study, we utilize ab initio molecular dynamics to investigate three types of betaine-amino acid based NaDES (Bet-AA), where betaine functions as a HBA and lysine, arginine, and histidine act as HBD, all in an equimolar ratio. The choice of these specific amino acids is due to their ability to form strong and complex noncovalent interactions, which are crucial to the microstructure and properties of NaDES. Expanding the range of amino acids with different polarities and hydrogen bonding propensities could significantly alter these interactions and thus modify the resulting physical and chemical properties of the solvents. Figure 1 illustrates the components of the electrolytes and their respective quantities.

The configurations of the NaDES in simulation box were generated randomly using Packmol software^15^ and simulated with classical full atomistic molecular dynamics simulations (CMD). Three systems were generated with AA molecules and betaine chloride ionic pairs into a cubic simulation box. The computational cells for AIMD simulations were generated with 11, 13, and 12 AA molecules (with an equal number of ion pairs), totaling 517, 540, and 533 atoms within the box for arginine, histidine, and lysine-based solutions, respectively. After equilibration simulations, the final sizes of the cells were 1.750, 1.788, and 1.770 nm for BetCl-Arg, BetCl-Hist, and BetCl-Lys DESs, respectively. All initial configurations were thermodynamically equilibrated through NPT-CMD at 298 K for 10 ns. The interactions between all atoms were modeled with OPLS-AA force field,^16^ implemented in version 2023.1 of the GROMACS software.^17,18^

AIMD was used to examine the local structures, complex network of intense noncovalent electrostatic interactions, and spectroscopic properties of the solutions with electronic precision. We use QUICKSTEP^19^ module within the CP2K software package^20^ for all quantum molecular dynamics. The exchange and correlation functional used to obtain the electronic interactions during the quantum simulations was BLYP.^21,22^ Additionally, we used the Grimme empirical dispersion correction (DFT-D3).^23^ The system was studied using the BLYP Goedecker-Teter-Hutter (GTH)^24,25^ pseudopotential set with the MOLOPT-DZVP-SR-GTH basis function set.^26^ The computational details also highlight a plane wave cutoff of 350 Ry, with 5 multigrids and a relative cutoff of 40 Ry, and a target precision of 10–6 Ha was also used for SCF convergence. All AIMD quantum simulations went through steps that use the canonical ensemble with Nosé–Hoover thermostats for individual atoms, with a time constant of 50 fs and a d*t* = 0.5 fs. Thus, two steps were performed: (a) step 1 – equilibration, for an equilibration period of 5 ps; and (b) step 2 – production, for 40 ps, totaling 90,000 time steps per run. We emphasize that all configurations were saved to ensure a detailed analysis. Finally, to calculate the cohesive energies of the electrolyte, we also performed gas phase simulations with the isolated species. All AIMD trajectories analyses were performed using the TRAVIS program.^27^

## 3 Results and Discussion

In this study, we initially performed an analysis of how AAs are involved in the thermal stability of NaDESs, for this we observed the cohesive energy density (*E*_coh_ = Δ*E*/*V*), extracted from AIMD-simulations. We emphasize that for this information we must also have *V* as the volume of the simulation box and Δ*E* as the difference between the bulk energy and the combined energies of the AA molecules and the ion pairs in the gas phase (Δ*E* = *E*_bulk_–*n*_AA_*E*_AA_–*n*_ip_*E*_ip_). The cohesive energy densities for BetCl-Arg, BetCl-Hist, and BetCl-Lys NaDESs are found to be −857 ± 14, −787 ± 14, and −736 ± 13 kJ mol^–1^ nm^–3^ for the systems based on arginine, histidine, and lysine, respectively (the uncertainties represent the standard deviation). The observed values are consistent with the number of polar sites present in each of the liquids, with the highest number found in the BetCl-Arg system. Among the three systems, BetCl-Arg exhibits the most extensive network of intermolecular connections. Conversely, the BetCl-Lys system, due to its aliphatic chain, has a smaller network of electrostatic bonds, with one fewer electrostatic site compared to the histidine-based system and two fewer compared to the arginine-based system (no experimental results are available for direct comparison). However, these findings are consistent with our previous AIMD results for DESs based on choline chloride mixed with organic liquids containing butanediol isomers.^28^ A significant difference from the butanediol-based systems is that the AA-based systems exhibit a cohesive energy density that is substantially higher (up to 50% greater). This indicates a much more extensive and intense network of electrostatic interactions than those observed in choline chloride and butanediol DESs.

**Figure 2:**
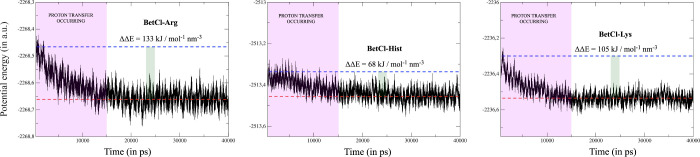
Potential energy as a function of time for all investigated NaDES. The colored band indicates the stage at which proton transfers occurred. The presented values represent the differences in cohesive energy densities before and after the initiation of the transfer process.

BetCl-AA-based NaDEs are characterized by a highly complex and dynamic three-dimensional hydrogen-bond (HB) network, which comprises a vast array of hydrogen-bonded and other noncovalent intermolecular interactions of various sizes and configurations. This intricate HB structure, with its strong cooperative nature, facilitates nearly barrier-free pathways for various dynamic processes such as the possible exchange of monomers in the liquid phase and an evident transfer of protons. After approximately 15 ps of evolution (see Figure 2), the system dynamics led to the rearrangement of the system’s electronic cloud and the consequent intra- and intermolecular proton transfers. These proton transfers (referring both to the deprotonation of betaine with its proton migrating to specific acceptor sites in each of the AA and a transfer of the proton from AA to its adjacent -NH_2_ terminus) stabilized the systems, reducing the cohesive energy density in 133, 68, and 105 kJ mol^–1^ nm^–3^, compared to the values before the deprotonation process began.

**Figure 3:**
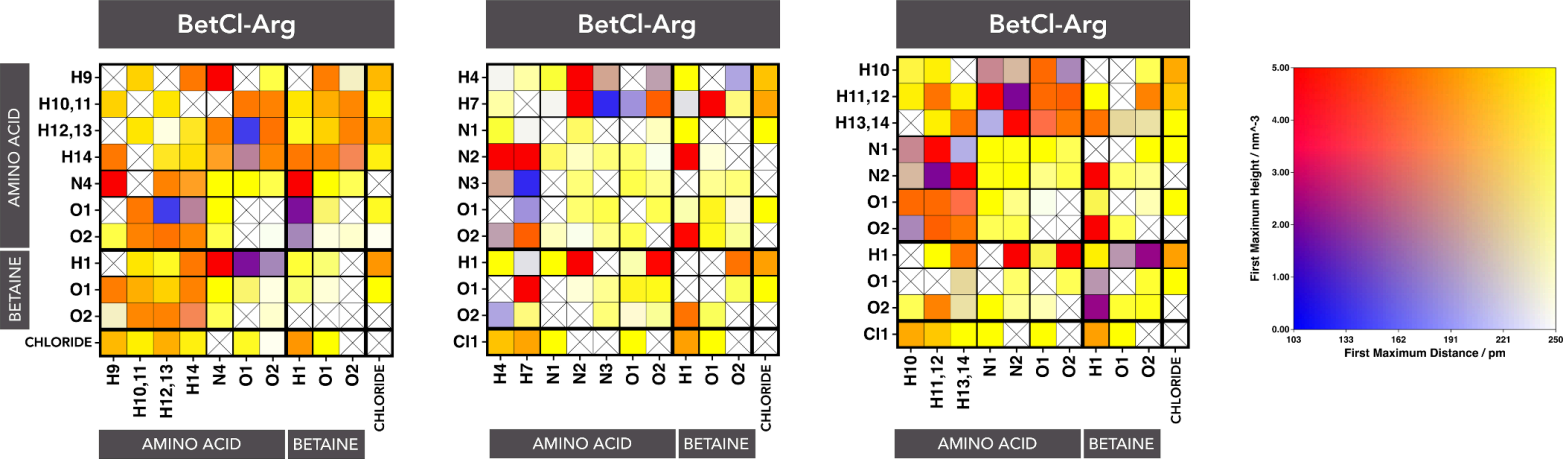
Connection matrices for the electrolyte components (amino acid, betaine cation, and chloride anion) in the three NaDESs analyzed. The colors represent the intensity and distance of the first peak in the radial distribution function (RDF), as shown in the 2D-colormap. The atom labels are presented in Figure 1.

The connection matrix can offer us a fundamental idea to understand how the network of interactions, basically noncovalent electrostatics, works in NaDES, which captures interactions among various HBD and HBA. Figure 3 presents the connection matrices, where all polar groups (acceptors and donors) as well as the chloride ion are represented on the vertical and horizontal axes. Nonpolar groups (carbon and hydrogen) are omitted since their contribution to electrostatic interactions is negligible or nonexistent. Each element marked with a cross indicates that the pair involved does not form a HB. Conversely, if a strong connection exists (normally a HB), the element will be colored according to an intensity scale shown on 2D-colormap of Figure 3.

By examining the matrices, it is evident that in all three cases, the AA molecules are involved in proton transfer processes. Proton transfers in AAs are well-documented and are linked to the isomerization of the neutral AA into its *zwitterionic* charge distribution, a process crucial for numerous biochemical functions.^29,30^ Such proton transfers are indicated by the red elements of the matrix, which are characterized by a very short distance (∼100 ps) and a very high peak (>30 nm^–3^) in the corresponding radial distribution function (RDF). Orange elements indicate HB contacts characterized by short distances (160–200 pm) and high to moderate peaks (2–5 nm^–3^). Yellow elements, in turn, indicate contacts with large maximum heights but also with large distances, and therefore represent less intense bonds.

The proton transfers observed in this simulation are clearly due to the quantum nature of the treatment applied to the electronic density, allowing for a rigorous study of the electronic density and the impact of its polarization on the HB network structure. In BetCl-Arg, for instance, we observe that proton from the carboxyl group, both of arginine (H9) or betaine (H1), is transferred to the nitrogen of another arginine (N4). Similar transfers are observed for the histidine-based solvent (BetCl-Hist), between the proton acceptor of histidine and the proton donors of histidine (H4 and H7) and betaine (H1). In the case of histidine, we remember that in the isolated phase, there are possible isomers for this amino acid; the second one being the isomer where the hydrogen H4 is covalently bonded to the nitrogen N3. This characteristic has been well discussed in the literature, and it seems that the initial protonation state (H4–N1) favors the migration of the hydrogen H4 to the nitrogen N2. For the BetCl-Lys, proton transfer primarily occurs between the proton-accepting nitrogen N1 and N2 of lysine and the proton donors of lysine (H11–H14) and betaine (H1).

The vast network of intense noncovalent bonds, both between the AA molecules and between AA and the betaine cation, is observed for all NaDES. However, for the BetCl-Hist, this network is significantly less intense. This is evident in its contact matrix (see Figure 3), which clearly shows a smaller number of orange elements in the contact matrix. The extent of these connection networks and the intensity of the bonds are strongly associated with the dynamic behavior of the liquids; this bonding network is responsible for the extremely high viscosity and very low ionic conductivity of these liquids.^7^

**Figure 4:**
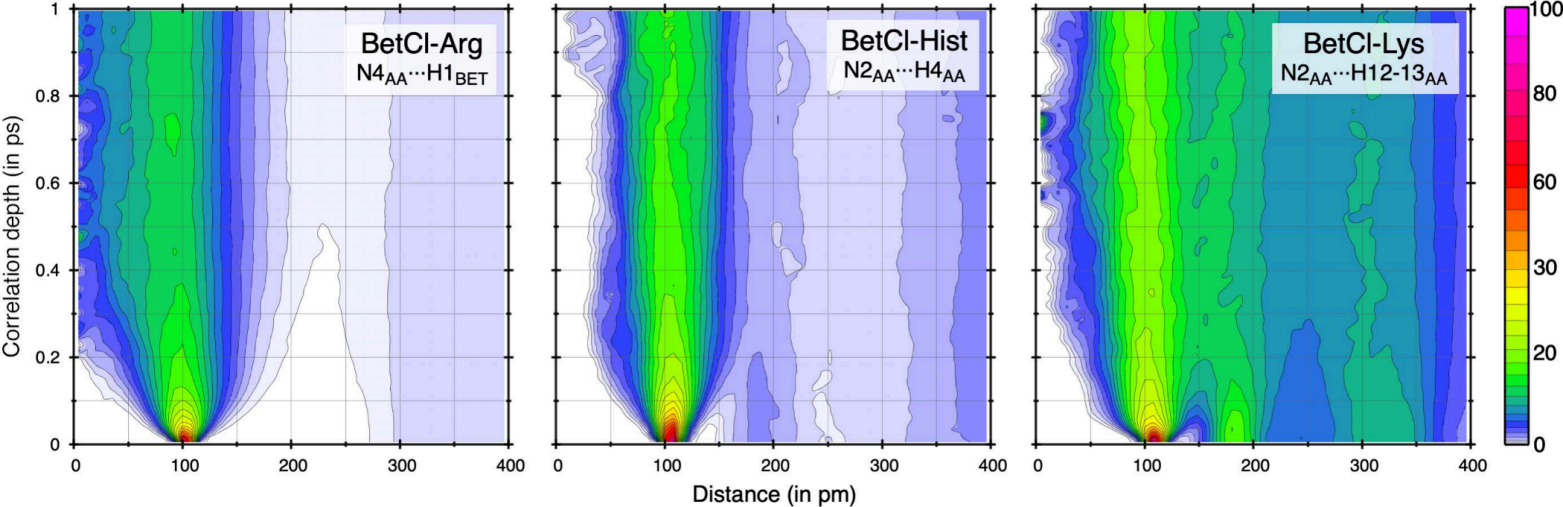
Van Hove correlation function (VHF) for the selected distances characterizing the dynamics of proton transfer for each investigated system.

Various sites, both from the cation and the AAs, are involved in proton transfers. In addition to the H1 and O2 atoms of the betaine cation, which participate in intermolecular proton transfers, specific sites of arginine (H9, N4, and H12–13), histidine (N2, H7, O2), and lysine (N1, N2, O2, H11, H12, H13, H14) also participate in both intermolecular and intramolecular transfers. The dynamics of these proton transfers can be investigated through temporal and spatial correlation functions, also known as Van Hove functions (VHF).^31^ VHF is a powerful tool in analyzing molecular dynamics simulations and characterizing intermolecular interactions, such as proton transfer and HBs formation. The VHF offers an in-depth explanation of how the position of a particle at one moment is related to the position of another particle at a subsequent time. This is particularly useful for studying rapid dynamic processes, such as proton transfer, where the location of a particle can change significantly over short periods. A VHF examines the positions of two particles across different trajectory frames, factoring in the correlation depth (τ) between them. In contrast, an RDF (refer to the discussion below) focuses on the distances between particles within the same trajectory’s configurations, aggregating these distances into a histogram. This allows for the analysis of the temporal evolution of intermolecular interactions. In the VHF, the standard RDF is reproduced at τ = 0, however, its features rapidly become less distinct as the correlation time increases, indicating that the observed peak reduces its intensity as τ increases. This behavior indicates the mobility of the particles and the efficiency of proton transfer or the formation and dissociation of HBs. In Figure 4, a VHF is presented for a selected atomic pair where a proton transfer was observed. The height of the first peak for each case, around 100–110 pm, is elevated at τ = 0, but it rapidly decays for longer times, reflecting the fast and dynamic nature of these transfers. This behavior is consistent with the nature of HBs, which are characterized by constant formation and breaking, facilitating processes such as proton transfer.

**Figure 5:**
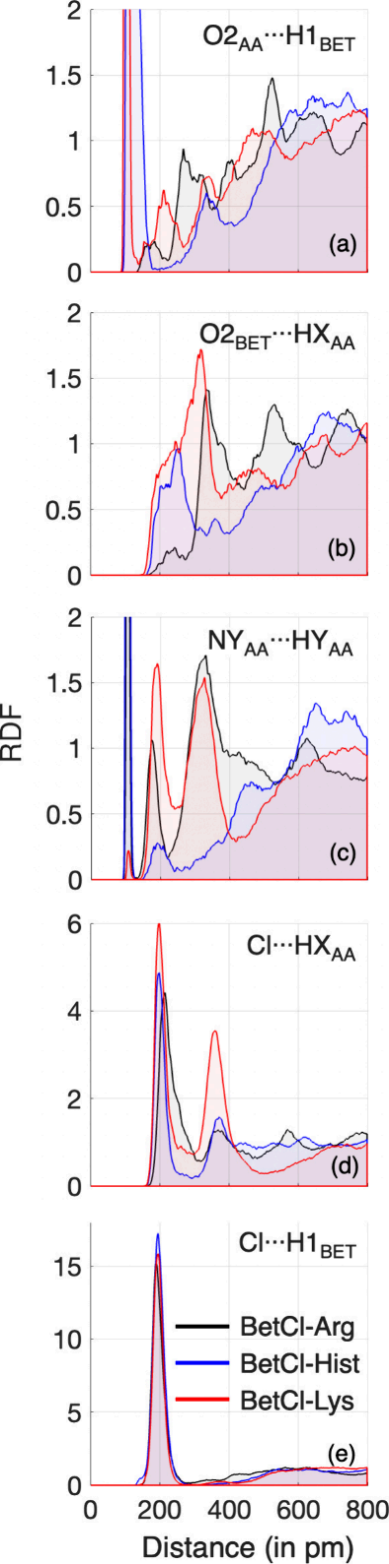
Some radial distribution functions highlighting noncovalent electrostatic atomic interactions. HY_AA_ are the hydrogen atoms of the amine group adjacent to the hydroxyl group. NY_AA_ are the amino acid terminal hydrogen atoms. HX_AA_ are the hydrogen atoms of the carboxyl group, involved in proton transfer.

In Figure 5, we present some RDFs for specific atomic interactions within DESs, especially H···O and H···Cl. Figure 5a,b illustrates the H···O RDFs between the AAs and the betaine cation, showcasing the typical characteristics observed in hydrogen-bonded systems. Both RDFs, O_AA_···H_BET_ and O_BET_···H_AA_, are similar except for the pronounced first peak of the former (Figure 5a), associated with the intramolecular H1_BET_···O2_AA_ bond, with the peak maximum located at ∼100 pm. The subsequent peaks, especially the second ones, describe the expected noncovalent hydrogen bonding. The RDFs in Figure 5c describe the mutual interactions between AAs, where the nitrogen atoms at the opposite end of the AA (NY_AA_: namely N4 in arginine, N3 in histidine, and N2 in lysine) interact with the hydrogens from the amine group from another AA molecule (HY_AA_). Again, the pattern repeats, with the presence of the first peak (which is very pronounced except in the BetCl-Lys system) attributed to proton transfer that enables the formation of an intramolecular N_AA_···H_AA_ bond, with the peak maximum located at ∼108 pm. Similarly, the subsequent peaks describe noncovalent bonding between these sites on the AA molecules.

Finally, we can also note that there is a significant role for the noncovalent electrostatic interactions that are formed between the chloride anion and the proton donor sites (Cl···H_BET_ and Cl···H_AA_). The intense coordination between these two sites (see Figure 5d,e) highlights the importance of these interactions in the structure of AA-based NaDES, particularly the strong coordination between the betaine cation and chloride anions, evidenced by the prominent first peak in Figures 5e. This type of interaction between chloride and cations has been previously observed in eutectic liquids, both experimentally,^32^ using IR and NMR spectroscopy, and computationally,^28^ through AIMD simulations, in chloride-based and choline-butanediol eutectic solvents.

**Figure 6:**
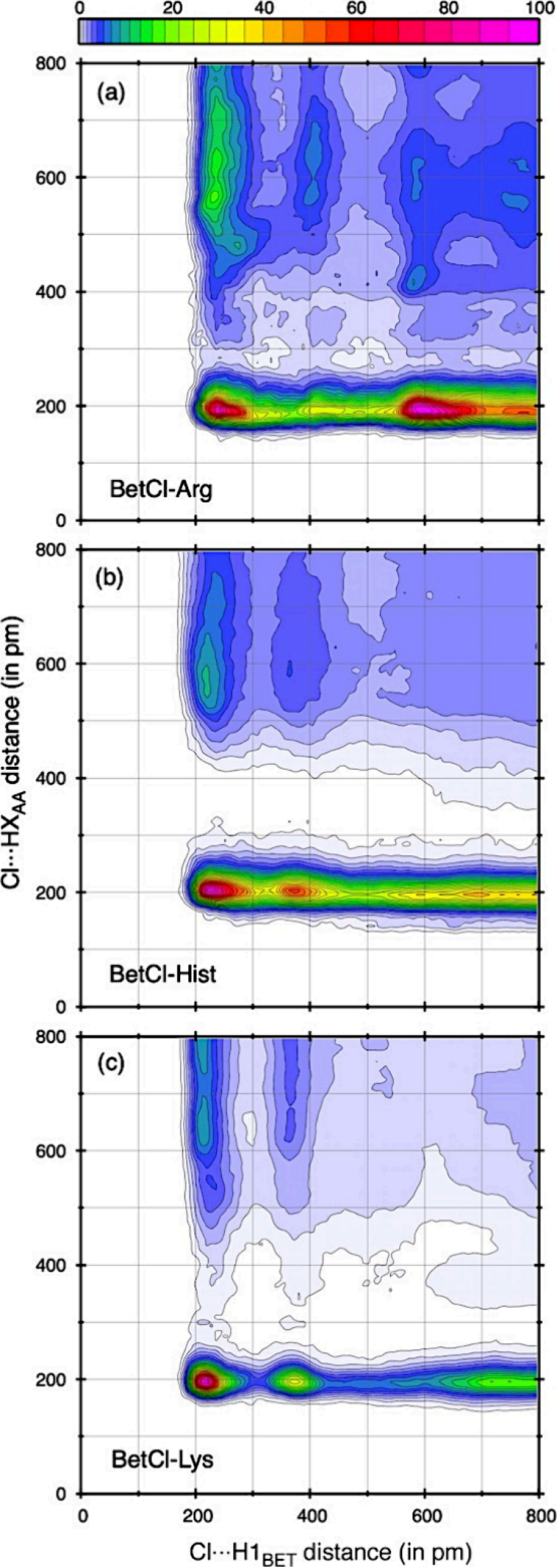
Correlation between the intermolecular distances involving Cl···H_BET_ and Cl···H_AA_ obtained from the Combined Distribution Functions (CDF). The tree plots are normalized to high maximum of 100 nm^–3^. HX_AA_ stands for atoms H10–11 at arginine, H8–9 at histidine and H11–12 at lysine.

A more comprehensive insight into how noncovalent electrostatic interactions involving the chloride anion affect the structure of NaDESs can be achieved by analyzing the combined distribution function (CDF, see Figure 6). This function illustrates the relationships between the distances of two different bonds. In this case, we simultaneously investigate the distributions involving chloride anion and: (a) H atoms of the acceptor sites of the betaine cation (Cl···H_BET_), and (b) AA molecule (Cl···H_AA_). Through the maps, we clearly observe that for both types of interaction, the greatest intensity, those closest, occur at essentially the same distance. The secondary peaks, in turn, are much less intense and, in the case of Cl···H_AA_ interactions, completely disconnected from the initial corresponding peaks. We therefore see that the anion–cation interaction network is altered by the presence of AAs, which replace part of these bonds with weaker ones; this is especially true for the BetCl-Arg system (Figure 6a), where this network of interactions shows its effects even at longer distances.

**Figure 7:**
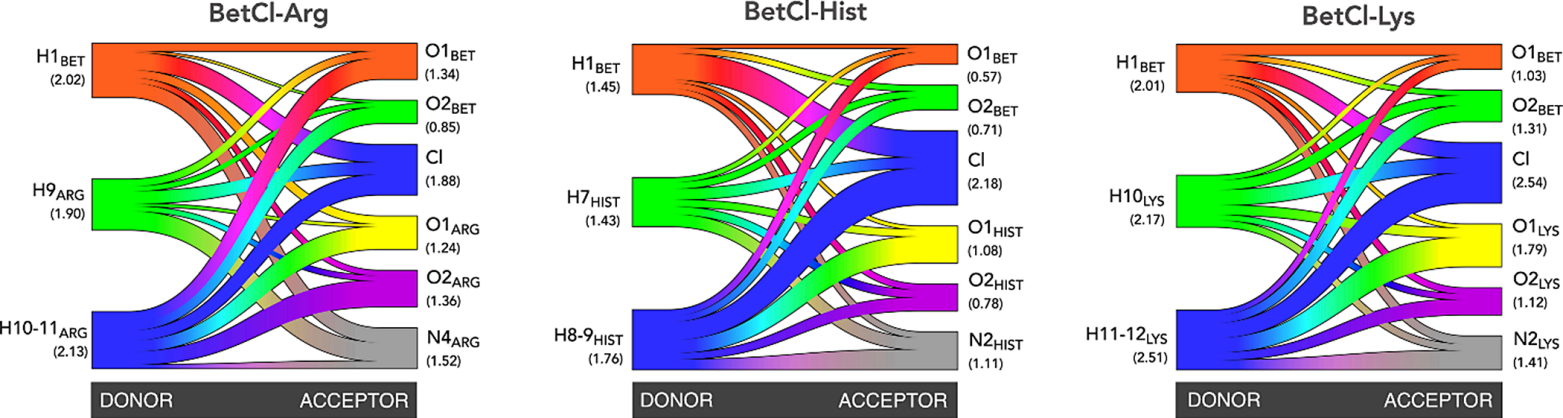
Sankey diagrams summarizing the HBs-topology and H···Cl contacts for the NaDES. In parentheses we shown the average #HB per atom. The first minimum of the RDF was used to define the contacts H···O and H···Cl. For atom labels, see Figure 3.

To better understand how the electrostatic interaction network is established in each NaDES can be obtained by examining its Sankey diagram^27^ (Figure 7), which allows for a detailed comprehension of the HB network topology in these systems, which are characterized by a type of electrical competition between proton donor and acceptor sites. In the Sankey diagram, the HBs are positioned on the left side (proton donors) and the O and Cl atoms are positioned on the right side (proton acceptors). The greater the number of HBs formed between the left and right components, the greater the width of the bands connecting the groups. Thus, we present in Figure 7 the three NaDES analyzed in this study and we can observe that the band widths are proportional to the average amount of HBs established by donor or acceptor atom (average number of HBs highlighted in parentheses). Therefore, the differences in the widths of each of these bands, connecting the left and right sides of the diagrams, indicate different molecule counts. Visually, it is possible to gauge the high complexity of the noncovalent interaction network in this type of system, which is qualitatively similar across the three NaDES, though there are significant quantitative differences. Quantitatively, each BetCl/AA pair forms, on average, 14.2, 11.1, and 15.9 bonds, respectively, in BetCl-Arg, BetCl-Hist, and BetCl-Lys, with approximately 80% of the bonds involving the AA molecule in each case. The average number of hydrogen bonds plays a crucial role in both the structural stability and the transport properties of NaDES. The greater the number of hydrogen bonds present, the more stable and interconnected the molecular network within the solvents becomes. Also, the large number of hydrogen bonds, particularly in BetCl-Arg (14.2) and BetCl-Lys (15.9), contributes to the high viscosity that is typically observed in these systems, since a denser hydrogen bond network restricts molecular mobility, leading to slower diffusion and reduced fluidity. For all three NaDESs, the pairs with the highest number of noncovalent electrostatic bonds are those formed by proton donors and the chloride anion (for the BetCl-Lys system, for example, chloride receives 2.54 bonds), which reinforces the essentiality of these interactions for the structure of NaDES. In particular, for the BetCl-Lys system, chloride receives 2.54 bonds from the proton donors of both the cation and the AAs. It should also be noted that there is a relatively large amount of HBs formed by HX_AA_ with donor sites from both betaine and neighboring AAs. This reinforces that the HB-topology of the noncovalent electrostatic network has a dependence between the amino acid molecules.

## 4 Conclusions

This study deep analysis of the interactions within natural deep eutectic solvents based on betaine chloride and arginine, histidine, and lysine amino acids (AA), utilizing ab initio molecular dynamics (AIMD) to unravel their complex structural and dynamic properties. The AIMD simulations demonstrated significant proton transfer activity within the NaDES. Proton transfers were observed between AA molecules and betaine cations, and among AAs themselves, contributing to the dynamic and complex nature of these solvents. The van Hove correlation functions (VHF) underscored the rapid and extensive proton transfers, indicating nearly barrier-free pathways for these processes. The radial distribution functions (RDF) illustrated a dense and dynamic hydrogen bond (HB) network. Both inter- and intramolecular HBs were prevalent, with chloride anions playing a critical role in maintaining this network by forming strong electrostatic interactions with proton donors. Our results also emphasized the critical role of chloride anion interactions with proton donors, demonstrating their significance in maintaining the structural framework of NaDES. The connection matrices and combined distribution functions (CDF) provided a detailed depiction of these interactions, revealing the nuanced balance between betaine and AA contributions to the HB network.

Different AAs influenced the structure and dynamics of the NaDES differently. Arginine-based NaDES exhibited the most extensive network of intermolecular connections, while lysine-based systems showed fewer, less intense interactions due to their aliphatic chain structure. The structural differences among the amino acids influence the hydrogen bonding network in NaDES by affecting both the number and type of hydrogen bonds formed. Arginine, with its guanidinium group, and lysine, with its terminal amine group, form a denser hydrogen bond network compared to histidine, due to their ability to engage in more proton donor and acceptor interactions. The dense HB network and significant proton transfers contribute to low ionic conductivity and the high viscosity of these NaDES. The intensity and number of HBs, especially involving chloride anions, were directly correlated with these physical properties. In conclusion, this study enhances the understanding of the structural/dynamic properties of betaine chloride-based NaDES. In summary, the findings from our research not only contribute to the fundamental understanding of NaDES but also offer practical insights that can be harnessed to enhance reaction efficiency, improve drug solubility, and promote sustainable practices in various industrial applications. By emphasizing these connections, we aim to position NaDES as a pivotal component in advancing biocatalytic methods, pharmaceutical formulations, and environmentally friendly chemical processes.
